# The importance of the lepidic component as a prognostic factor in stage I pulmonary adenocarcinoma

**DOI:** 10.1186/s12957-016-0791-y

**Published:** 2016-02-16

**Authors:** Youngkyu Moon, Sook Whan Sung, Kyo Young Lee, Young Kyoon Kim, Jae Kil Park

**Affiliations:** Department of Thoracic & Cardiovascular Surgery, Seoul St. Mary’s Hospital, College of Medicine, The Catholic University of Korea, 222 Banpo-daero, Seocho-gu, Seoul, 06591 Republic of Korea; Department of Hospital Pathology, Seoul St. Mary’s Hospital, College of Medicine, The Catholic University of Korea, Seoul, Republic of Korea; Department of Internal Medicine, Seoul St. Mary’s Hospital, College of Medicine, The Catholic University of Korea, Seoul, Republic of Korea

**Keywords:** Lung cancer, Adenocarcinoma, Lepidic, Prognosis, Stage I, Recurrence, Adenocarcinoma classification

## Abstract

**Background:**

Stage I pulmonary adenocarcinoma (PA) can offer an unfavorable prognosis. The aim of this study was to classify the prognosis of stage I PA on the basis of the lepidic component and to confirm whether the lepidic component can be used as a criterion for predicting the prognosis of stage I PA.

**Methods:**

We conducted a retrospective study of patients who underwent curative surgery for stage I and IIA PA. Stage I disease was divided into three groups on the basis of the lepidic component: group 1, ≤10 %; group 2, >10 to 50 %; and group 3, >50 %. We compared recurrence-free survival (RFS) rates among groups 1, 2, and 3, and stage IIA disease. We also evaluated risk factors for disease recurrence with multivariate analysis.

**Results:**

A total of 224 patients were included in our study; most patients (*n* = 201) had stage I disease. Three-year RFS rates in group 1 (*n* = 73), group 2 (*n* = 75), and group 3 (*n* = 53) were 74.1, 90.4, and 90.0 %, respectively. There was a significant difference in RFS between group 1 and group 2 (*p* = 0.009). The 3-year RFS rate in stage IIA disease was 61.4 %. There were no significant differences in RFS between group 1 and stage IIA disease (*p* = 0.163). In multivariate analysis, group 1 had the highest risk of recurrence (HR 5.806, *p* = 0.006) in stage I PA.

**Conclusions:**

Stage I PA with a lepidic component ≤10 % was associated with an unfavorable prognosis that was similar to the prognosis of stage IIA disease. The prognosis for stage I PA should not be based on general criteria, but instead, the lepidic component should be evaluated and considered when determining disease prognosis.

## Background

Pulmonary adenocarcinoma (PA) is the most common histologic type of primary lung cancer [1]. In 2011, the International Association for the Study of Lung Cancer (IASLC), the American Thoracic Society (ATS), and the European Respiratory Society (ERS) proposed new subtypes of adenocarcinoma. According to the new classification system, the components of adenocarcinoma were classified into five major histopathologic categories on the basis of the growth pattern or shape of the tumor: acinar, papillary, micropapillary, solid, and lepidic [2]. Among the subtypes of PA, lepidic-predominant adenocarcinoma has the most favorable prognosis, and micropapillary-predominant and solid-predominant adenocarcinomas have unfavorable prognoses [2, 3]. However, in more than 90 % of PA cases, two or more components are present and the prognosis of PA is complicated and varies on the basis of the ratio of each histopathologic component. After the adoption of the new classification system, many studies reported correlations between subtypes and histopathologic components of PA and disease prognosis [3–8].

Until now, the most important prognostic factor in non-small cell lung cancer (NSCLC) has been its anatomical stage [9]. However, recurrence can occur even with the most favorable prognosis in stage I, which indicates that other factors may affect prognosis and that the prognosis of stage I disease varies with histopathologic subtype [10]. Additionally, one study reported that the prognosis could vary with the respective occupancy rates of the five histopathologic components [5]. Therefore, stage I PA may be able to be divided into favorable prognosis and unfavorable prognosis, which would help to predict the disease course for patients with stage I PA. The lepidic component is the most common component among the five histopathologic components in stage I PA, so it is reasonable to classify stage I PA on the basis of the occupancy rate of the lepidic component.

The aim of this study was to confirm the usefulness of classifying the prognosis of stage I PA on the basis of the occupancy rate of the lepidic component. Further, we aimed to evaluate whether the lepidic component could be used to predict the prognosis of PA.

## Methods

### Patients

We conducted a retrospective chart review of 265 patients who were diagnosed with stage I and stage IIA PA and underwent curative resection at Seoul St. Mary’s Hospital at the Catholic University of Korea between August 2010 and December 2013. Among 242 patients with stage I PA, 41 patients were excluded from the study: 25 patients who were diagnosed with adenocarcinoma in situ (AIS) or minimally invasive adenocarcinoma (MIA), 15 patients who were diagnosed with mucinous adenocarcinoma, and 1 patient who was diagnosed with adenocarcinoma with neuroendocrine features. Mucinous adenocarcinoma was excluded from our study because it exhibits clinical, pathologic, and genetic differences from non-mucinous adenocarcinoma and it is classified in a new category of “variants of invasive adenocarcinoma” in the IASLC/ATS/ERS classification system [2]. A total of 224 patients were included in the final study analysis: 201 patients with stage I PA and 23 patients with stage IIA PA. Cancer staging was determined according to the TNM classification system defined by the American Joint Committee on Cancer [9]. This study was approved by the institutional review board of Seoul St. Mary’s Hospital at the Catholic University of Korea.

### Histologic evaluation

All clinical specimens were examined by pathology specialists, and their observations were recorded in pathology records. For the description of histopathologic components, the occupancy ratio of each component (lepidic, acinar, papillary, micropapillary, and solid) in the total tumor area was measured and recorded in 5 % increments, according to the 2011 IASLC/ATS/ERS classification system [2]. The 201 patients with stage I disease were divided into three groups on the basis of the occupancy ratio of the lepidic component in the total tumor: group 1, ≤10 %; group 2, >10 to 50 %; and group 3, >50 %.

### Statistical analysis

We divided stage I disease into three groups according to the percentage of the lepidic component, and we compared the clinicopathologic characteristics among the groups. We also compared characteristics between stage I and stage IIA disease and group 1 and stage IIA disease. For these comparisons, we used Student’s *t* test for continuous variables and the *χ*^2^ test for categorical variables. We used the analysis of variance test and the Kruskal-Wallis H test to compare the means of the groups. We analyzed data from the time of surgery to the last date of follow-up, and we determined recurrence-free survival (RFS) using the Kaplan-Meier method by calculating the cases of recurrence and death. We compared survival rates among the groups using log rank statistics. We used the Cox proportional hazards model for the multivariate analysis to determine risk factors for recurrence in patients with stage I disease. A value of *p* < 0.05 was considered statistically significant.

## Results

The mean age of all 224 patients (stages I and IIA) was 63.7 years (range, 38–85 years), and more than half (58.9 %) of the patients were female. Stage I disease was divided into groups 1, 2, and 3 according to the lepidic component; the clinical characteristics of each group are presented in Table 1. Group 1 contained more male patients (60.3 %) than female patients, but groups 2 and 3 contained more female patients than male patients (*p* < 0.001). Mean smoking amount of group 1 was significantly higher than that of group 2 but was not significantly different from that of group 3. There were no differences in age, sex, or smoking history between patients with stage I and stage IIA disease. The maximum standardized uptake value (SUVmax) of fluorodeoxyglucose in positron emission tomography (PET) in group 1 was significantly higher than that of group 3 but was not different than that of group 2. The SUVmax in stage IIA disease was significantly higher than that in stage I disease (*p* < 0.001). The SUVmax in stage IIA disease was also significantly higher than that in group 1 (8.8 vs. 4.5; *p* < 0.001). There was no difference in the rate of pulmonary resection among groups 1, 2, and 3 or between stage I and stage IIA disease. Mediastinal lymph node dissection or sampling was conducted for every patient; there were no differences in the average number of removed lymph nodes among groups 1, 2, and 3, but there was a small difference between stage I and IIA disease (13.5 ± 9.5 vs. 17.7 ± 8.5, respectively; *p* = 0.043).

**Table 1:** Clinical characteristics of patients with pulmonary adenocarcinoma, according to disease stage and lepidic component

	Stage I					Stage IIA	*p* value^b^
	Total	Group 1	Group 2	Group 3	*p* value^a^	(*n* = 23)	
	(*n* = 217)	(*n* = 73)	(*n* = 75)	(*n* = 53)			
Age, years (±SD)	64.0 (±9.3)	64.5 (±10.3)	65.2 (±9.4)	61.8 (±7.4)	0.121	61.0 (±7.5)	0.131
Sex					<0.001		0.518
Female, *n* (%)	117 (58.2 %)	29 (39.7 %)	59(78.7 %)	29(54.7 %)		15 (65.2)	
Male, *n* (%)	84 (41.8 %)	44 (60.3 %)	16 (21.3 %)	24 (45.3 %)		8 (34.8)	
Smoking, pack years (±SD)	8.6 (±15.6)	14.2 (±19.1)	3.8 (±10.9)	7.3 (±13.3)	<0.001	5.7 (±14.7)	0.424
Laterality					0.469		0.038
Right, *n* (%)	137 (68.2 %)	47 (64.4 %)	55 (73.3 %)	35 (66.0 %)		10 (43.5 %)	
Left, *n* (%)	64 (31.8 %)	26 (35.6 %)	20 (26.7 %)	18 (34.0 %)		13 (56.5 %)	
SUVmax (±SD)	3.6 (±3.2)	4.5 (±3.8)	3.7 (±3.0)	2.0 (±1.8)	<0.001	8.8 (±3.7)	<0.001
Extent of operation					0.416		0.273
Wedge resection, *n* (%)	17 (8.5 %)	8 (11.0 %)	5 (6.7 %)	4 (7.5 %)		0	
Segmentectomy, *n* (%)	10 (5.0 %)	2 (2.7 %)	6 (8.0 %)	2 (3.8 %)		0	
Lobectomy, *n* (%)	170 (84.6 %)	62 (84.9 %)	61 (81.3 %)	47 (88.7 %)		22 (95.7 %)	
Bilobectomy, *n* (%)	4 (2.0 %)	1 (1.4 %)	3 (4.0 %)	0		1 (4.3 %)	
Surgical approach					0.475		0.692
VATS, *n* (%)	178 (88.6 %)	62 (84.9 %)	68 (90.7 %)	48 (90.6 %)		21 (91.3 %)	
Thoracotomy, *n* (%)	21 (11.4 %)	11 (15.1 %)	7 (9.3 %)	5 (9.4 %)		2 (8.7 %)	
Sampled lymph node, *n* (±SD)	13.5 (±9.5)	14.0 (±10.7)	14.0 (±9.2)	12.3(±7.9)	0.537	17.7 (±8.5)	0.043

Group 1 = lepidic component ≤10 %; group 2 = lepidic component >10 to 50 %; group 3 = lepidic component >50 %

*SD* standard deviation, *SUVmax* maximum standardized uptake value of fluorodeoxyglucose, *VATS* video-assisted thoracoscopic surgery

^a^Comparison among groups 1, 2, and 3

^b^Comparison between stage I and stage IIA

The pathologic characteristics of patients with stage I PA, according to the lepidic component, are presented in Table 2. There was no difference in average tumor size (*p* = 0.076). The incidence of pleural invasion and vascular invasion were higher in groups 1 and 2 than in group 3. The occupancy ratios of micropapillary and acinar components were similar in groups 1 and 2, and the occupancy ratios of solid and papillary components were significantly higher in group 1 than in groups 2 and 3.

**Table 2:** Pathologic characteristics of patients with stage I pulmonary adenocarcinoma according to lepidic component

	Group 1	Group 2	Group 3	*p* value
Tumor size, cm (±SD)	2.2 (±0.9) cm	2.3 (±0.9) cm	2.0 (±0.7) cm	0.076
Pleural invasion, *n* (%)	23 (31.9 %)	18 (24.3 %)	4 (7.5 %)	0.005
Lymphatic invasion, *n* (%)	21 (29.2 %)	28 (37.8 %)	9 (17.0 %)	0.039
Vascular invasion, *n* (%)	9 (12.5 %)	9 (12.5 %)	1 (1.9 %)	0.088
Differentiation				<0.001
Mild, *n* (%)	15 (20.5 %)	41 (54.7 %)	48 (90.6 %)	
Moderate, *n* (%)	49 (67.1 %)	31 (41.3 %)	5 (9.4 %)	
Poor, *n* (%)	9 (12.3 %)	3 (4.0 %)	0 (0 %)	
EGFR mutation	75.4 %	90.3 %	79.4 %	0.087
Micropapillary component, % (±SD)	3.4 (±9.4)%	3.0 (±8.5)%	0.1 (±0.7)%	0.006
Solid component, % (±SD)	15.2 (±27.9)%	1.1 (±3.7)%	0.3 (±1.6)%	<0.001
Acinar component, % (±SD)	49.0 (±37.7)%	52.2 (±21.6)%	21.0 (±11.2)%	<0.001
Papillary component, % (±SD)	23.8 (±22.5)%	8.7 (±18.2)%	2.9 (±6.8)%	<0.001

Group 1 = lepidic component ≤10 %; group 2 = lepidic component >10 to 50 %; group 3 = lepidic component >50 %

*EGFR* epidermal growth factor receptor, *SD* standard deviation

The pathologic characteristics of patients in group 1 and patients with stage IIA disease are presented in Table 3. Patients with stage IIA disease had a larger tumor size than patients in group 1 (3.0 vs. 2.2 cm; *p* = 0.005), as well as higher rates of lymphatic invasion and vascular invasion (*p* < 0.001 and *p* = 0.001, respectively). However, there were no significant differences in the occupancy ratios of histopathologic components of the tumor between these two groups. Specifically, the lepidic component was not different between group 1 and stage IIA disease (4.4 vs. 9.9 %, respectively; *p* = 0.095).

**Table 3:** Comparison of pathologic characteristics between patients in group 1 and patients with stage IIA pulmonary adenocarcinoma

	Group 1	Stage IIA	*p* value
Tumor size, cm (±SD)	2.2 (±0.9)cm	3.0 (±1.3)cm	0.005
Pleural invasion, *n* (%)	23 (31.9 %)	9 (40.9 %)	0.298
Lymphatic invasion, *n* (%)	21 (29.2 %)	20 (87.0 %)	<0.001
Vascular invasion, *n* (%)	9 (12.5 %)	11 (47.8 %)	0.001
Differentiation			0.534
Mild, *n* (%)	15 (20.5 %)	4 (17.4 %)	
Moderate, *n* (%)	49 (67.1 %)	14 (60.9 %)	
Poor, *n* (%)	9 (12.3 %)	5 (21.7 %)	
EGFR mutation	75.4 %	76.2 %	0.597
Micropapillary component, % (±SD)	3.4 (±9.4)%	5.5 (±8.9)%	0.378
Solid component, % (±SD)	15.1 (±27.9)%	15.7 (±29.5)%	0.936
Acinar component, % (±SD)	49.0 (±37.7)%	48.3 (±28.5)%	0.927
Papillary component, % (±SD)	23.84 (±33.5)%	17.1 (±22.7)%	0.296
Lepidic component, % (±SD)	4.4 (±4.6)%	9.9 (±15.0)%	0.095

Group 1 = stage I disease with lepidic component ≤10 %

*EGFR* epidermal growth factor receptor, *SD* standard deviation

**Fig. 1:**
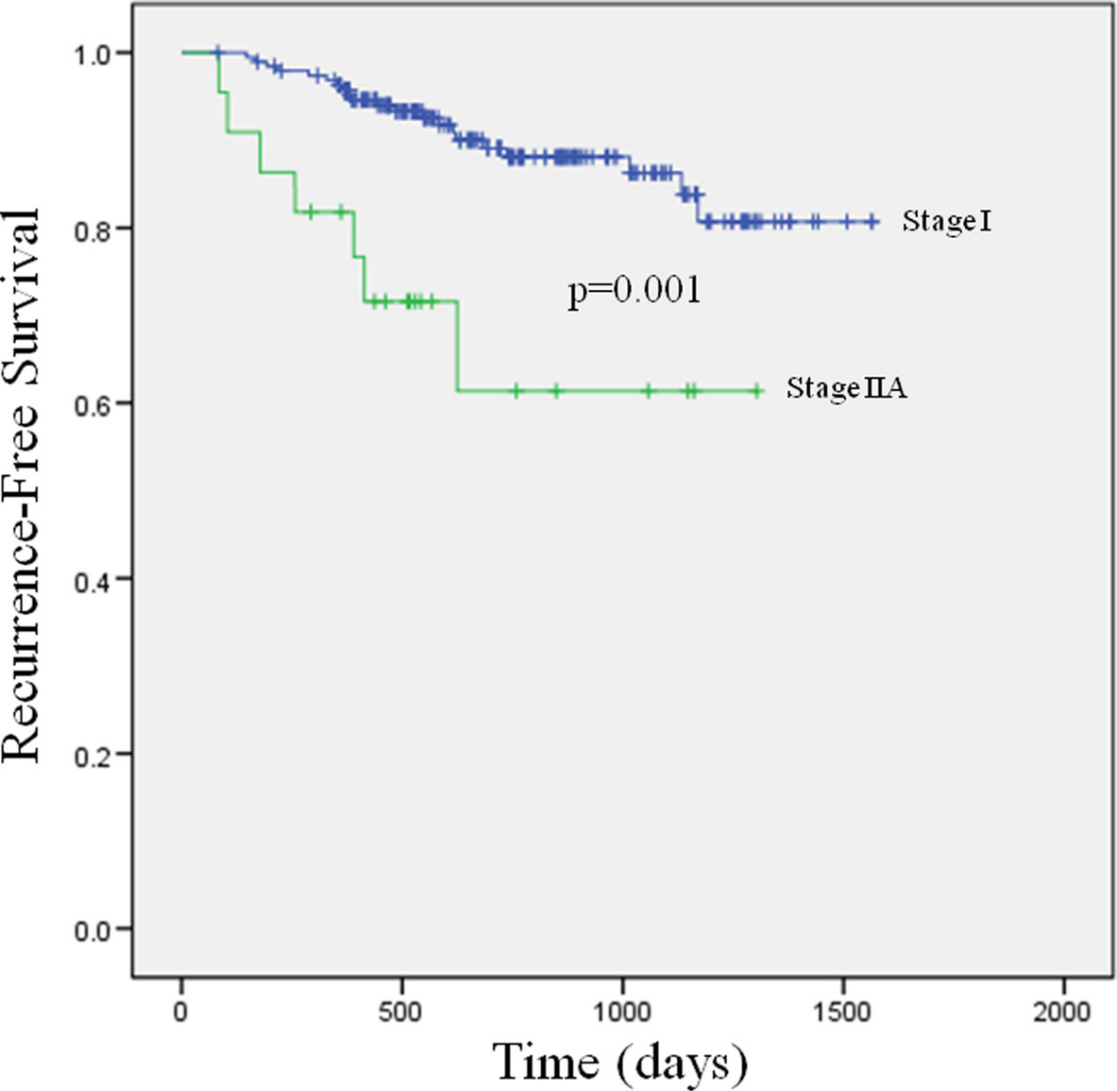
Three-year recurrence-free survival of patients with stage I and stage IIA pulmonary adenocarcinoma

**Fig. 2:**
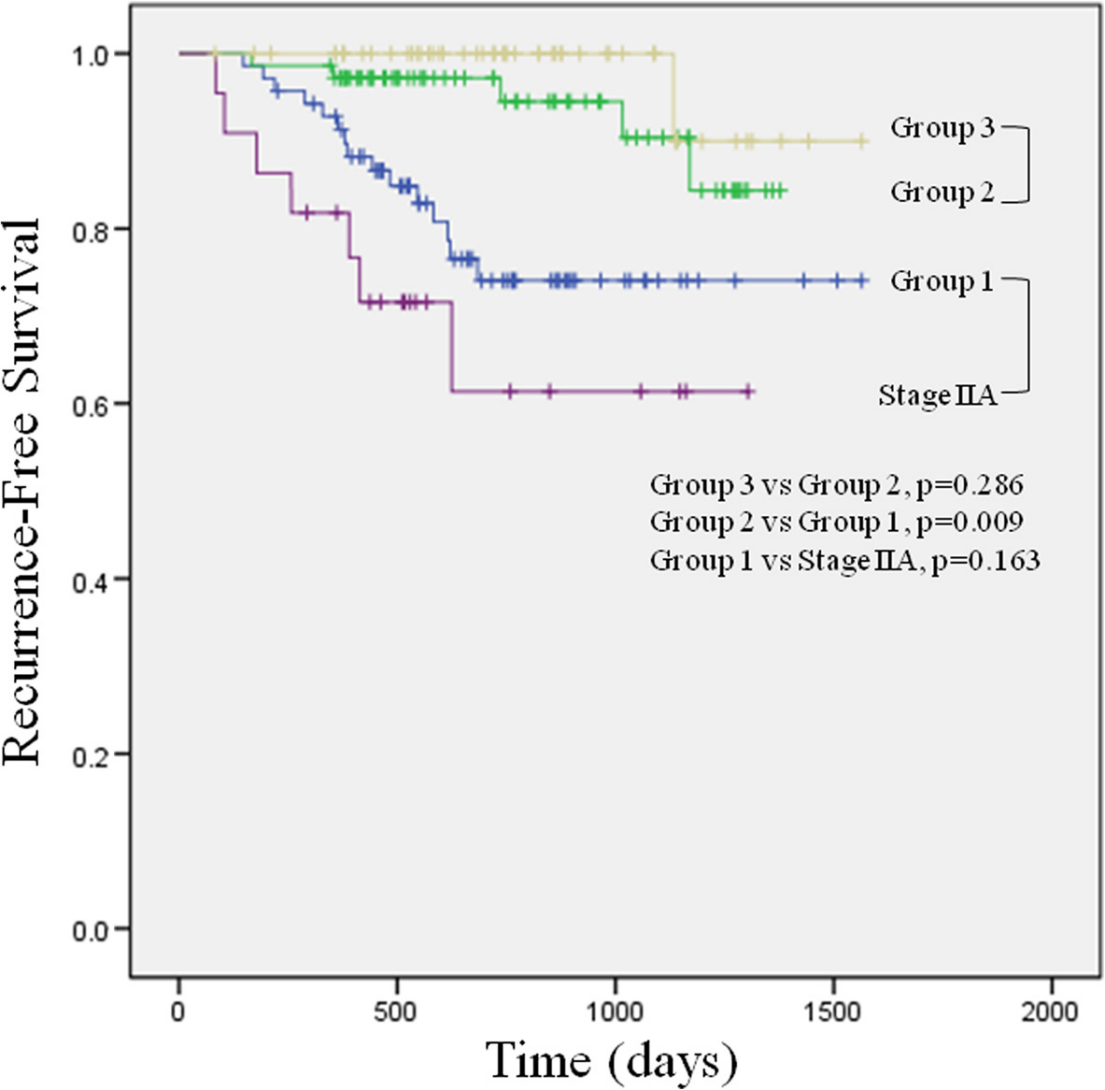
Three-year recurrence-free survival among three groups of patients with stage I pulmonary adenocarcinoma and patients with stage IIA pulmonary adenocarcinoma. Group 1 = stage I disease with lepidic component ≤10 %; group 2 = stage I disease with lepidic component >10 % to 50 %; group 3 = stage I disease with lepidic component >50 %

The median follow-up time for all patients was 720 days (range, 12–1564 days). During the follow-up period, disease recurrence occurred in 22 (10.9 %) patients with stage I PA and 7 (30 %) patients with stage IIA disease. The 3-year RFS rate in stage I disease was significantly higher than in stage IIA disease (86.3 vs. 61.4 %, respectively; *p* = 0.001) (Fig. 1). The 3-year RFS rates in groups 1, 2, and 3 were 74.1, 90.4, and 90.0 %, respectively (Fig. 2). The RFS in group 2 was significantly higher than that in group 1 (*p* = 0.009), but it was not different than that in group 3. There was no difference in 3-year RFS between group 1 and stage IIA disease (74.1 vs. 61.4 %, respectively; *p* = 0.163).

The risk factors for recurrence according to multivariate analysis are presented in Table 4. We analyzed the respective effect of each of the five histopathologic components (%) on disease recurrence using covariate factors of age, sex, smoking history, SUVmax, tumor size, and rates of pleural invasion, lymphatic invasion, and vascular invasion. Only the lepidic component was significantly related to recurrence (hazard ratio (HR) = 0.955, 95 % CI = 0.924–0.987; *p* = 0.007). A lepidic component of ≤10 % (group 1) was associated with the highest risk of recurrence (HR = 5.806, 95 % CI = 1.666–20.238; *p* = 0.006).

**Table 4:** Multivariate analysis of risk factors for recurrence of pulmonary adenocarcinoma

	HR	95 % CI	*p* value
Age	0.960	0.911–1.011	0.120
Sex	0.678	0.161–2.853	0.597
Smoking history	1.006	0.974–1.039	0.714
SUVmax	1.011	0.860–1.189	0.893
Tumor size	1.456	0.715–2.965	0.300
Pleural invasion	0.469	0.117–1.874	0.284
Lymphatic invasion	2.487	0.677–9.134	0.170
Vascular invasion	1.496	0.321–6.960	0.608
Lepidic component (%)	0.955	0.924–0.987	0.007
Micropapillary component (%)	1.040	0.991–1.092	0.114
Acinar component (%)	1.012	0.993–1.032	0.209
Papillary component (%)	1.016	0.994–1.038	0.155
Solid component (%)	0.982	0.927–1.041	0.546
Lepidic component ≤10 %	5.806	1.666–20.238	0.006

*CI* confidence interval, *HR* hazard ratio, *SUVmax* maximum standardized uptake value of fluorodeoxyglucose

## Discussion

Most cases of PA display a mixture of five histopathologic components. In the early stages of the disease, the lepidic component is the most common component. In our study, we divided stage I disease into three relatively even groups on the basis of lepidic component (*n* = 73, 75, and 53). The 3-year RFS rates of the three groups were 74.1, 90.4, and 90.0 %, and we demonstrated that a higher proportion of lepidic component in the tumor was associated with better survival. RFS was lowest with a lepidic component ≤10 %, and the prognosis for patients with stage I disease with a lepidic component ≤10 % was not better than the prognosis for patients with more advanced disease.

According to the 2011 IASLC/ATS/ERS classification criteria, acinar, papillary, micropapillary, and solid components are classified as invasive components and differentiated from lepidic components [2]. Therefore, a higher proportion of invasive components is associated with easier tumor invasion and an increased likelihood of tumor progression. Consequently, the relative amounts of components other than the lepidic component could be important factors in determining prognosis. In the case of stage I disease, the occupancy ratio of the lepidic component is so high that the occupancy ratios of the other components are relatively low. In our study, groups 1 and 2 showed different survival rates, and an evaluation of the different components present in the tumors of these groups indicated that only solid and papillary components were higher in group 1 and that there were no differences in the ratios of acinar or micropapillary components. Many studies have reported that only micropapillary and lepidic components are independently related to disease recurrence, and few studies have reported that acinar, solid, or papillary components individually affect prognosis [4, 5, 11, 12]. Since we observed no difference in the proportion of micropapillary component between groups 1 and 2, we conclude that the difference in survival was due to the difference in lepidic component and not a difference in solid or papillary components. Further, since the results of our multivariate analysis indicated that only the lepidic component was found to be a risk factor for recurrence, the low proportion of lepidic component could be emphasized as a significant risk factor for disease recurrence.

In NSCLC, stage IA and IB disease are differentiated by tumor size and the presence of visceral pleural invasion [9] and stage IA disease has a higher survival rate than stage IB disease [13–15]. However, in addition to tumor size and visceral pleural invasion that differentiate stages IA and IB, many other factors are being investigated that might influence disease prognosis, including histopathologic components. In our study, we included tumor size and visceral pleural invasion as covariate factors in the multivariate analysis and the percentage of lepidic component was found to influence prognosis. In group 1, specifically, a low percentage of lepidic component was determined to be a risk factor for recurrence with an HR of 5.8. Therefore, this criterion could be used to determine prognosis as well as differentiate stage IA from stage IB disease.

When we examined the clinicopathologic characteristics of group 1, we observed that this group contained more males and more people with a smoking history than groups 2 and 3, as well as stage IIA disease. It shows that the characteristics of patients in group 1 were different than those of patients with other types of adenocarcinoma. We observed no significant differences between groups 1 and 2 when we evaluated other known prognostic factors such as SUVmax, tumor size, percentage of micropapillary component, and rates of pleural invasion, lymphatic invasion, and vascular invasion [3, 16–20]. However, there was a difference in RFS between groups 1 and 2, which indicated that the difference in prognosis was due to the difference in the lepidic component between the groups.

The lepidic component represents only a small proportion of tumors in stage II disease, so it is only possible to differentiate stage I disease according to the lepidic component [5]. The results of our comparison of clinicopathologic characteristics between group 1 and stage IIA disease showed that there was significantly more lymphatic invasion and vascular invasion and tumor size was larger in stage IIA disease. Tumor size and lymphovascular invasion are important prognostic factors for PA [16–19]. SUVmax, which is a known prognostic factor in early lung cancer, was also significantly higher in stage IIA disease [3, 20]. Moreover, 21 (91.3 %) patients with stage IIA disease had lymph node metastasis. On the basis of these results alone, the prognosis of stage IIA could be assumed to be worse than that of group 1. However, we observed no difference in the distributions of histopathologic components or 3-year RFS between group 1 and stage IIA disease. Therefore, we inferred that the prognosis of stage I disease could be similar to that of stage IIA disease if the histopathologic components showed similar patterns, even if the pathologic features are worse in stage IIA disease.

In this study, we evaluated RFS instead of overall survival because, in the case of stage I disease, more patients die from other causes than from the relevant cancer during the follow-up period [3]. Also, RFS is a more accurate measurement of survival analysis, since it reflects the biological behavior of the cancer rather than death due to unrelated factors. Further, since we only included relatively recent data in our analysis, we were only able to use 3-year survival data to evaluate disease prognosis. This restriction was necessary because the institution at which the authors practice established video-assisted thoracoscopic surgery (VATS) as the general method of operation beginning in August 2010; since that time, this type of operation has been performed on the majority of patients at the institution and full mediastinal lymph node dissection has been performed. In addition, we determined that 3-year RFS was sufficient to examine prognosis since, in PA, most cases of postoperative recurrence occur within 2 years and cancer that recurs after 2 years has a high likelihood of being metachronous cancer [21].

This study has several limitations that should be considered. First, we used a retrospective study design. Second, we obtained the data from a single institution and the number of cases was relatively small. However, most studies that have evaluated the histopathologic classification of stage I PA included approximately 200 cases. Compared to these studies, the 217 patients with stage I disease included in our study do not comprise a small sample size [10, 22–27]. Of course, more accurate results could be obtained if the analysis and comparison were made with a larger patient sample. Third, we used recent data (2010 to 2013), so only short-term follow-up information was available and only 3-year survival evaluation was possible. However, as mentioned, most cases of recurrence of lung cancer occur within 2 years, and we judged that 3-year RFS was suitable for the determination of prognosis [21]. More accurate results could be obtained if more data were available from progressive observations over a longer period of time.

## Conclusions

In conclusion, recurrence often occurs in stage I PA, even after curative surgery. Specifically, recurrence is likely in cases in which the lepidic component of the tumor is less than or equal to 10 %. Further, in stage I disease with a low lepidic component, recurrence rates are not significantly different than those of stage IIA disease and generally applied stage I prognoses should not be expected. Adjuvant treatment, which is often performed in stage II disease, can be considered for stage I disease with a low lepidic component. Future studies that collect data from larger sample sizes will confirm the relationships between the histopathologic components of PA and disease prognosis.

## Abbreviations

ATS: American Thoracic Society
ERS: European Respiratory Society
HR: hazard ratio
IASLC: International Association for the Study of Lung Cancer
NSCLC: non-small cell lung cancer
PA: pulmonary adenocarcinoma
PET: positron emission tomography
RFS: recurrence-free survival
SUVmax: maximum standardized uptake value
VATS: video-assisted thoracoscopic surgery
